# Impact of the Smart Tablet Education for Healthy Living (STEHL) Intervention: Results from a Focus Group Study

**DOI:** 10.1093/geroni/igaf122.518

**Published:** 2025-12-31

**Authors:** Yujun Liu, Elizabeth Sterner, Bolanle Olayeni, Shalini Shrikanth, Pamela Gampetro, Rhea Johnson, Masooma Shamsi, Anitha Saravanan

**Affiliations:** Northern Illinois University, Naperville, Illinois, United States; Northern Illinois University, DeKalb, Illinois, United States; Northern Illinois University, DeKalb, Illinois, United States; Loyola University Chicago, Chicago, Illinois, United States; Illinois State University, Evanston, Illinois, United States; Northern Illinois University, DeKalb, Illinois, United States; Northern Illinois University, DeKalb, Illinois, United States; Northern Illinois University, DeKalb, Illinois, United States

## Abstract

**Background and Objectives:**

Older adult Americans often struggle with digital health literacy (DHL), the ability to find, evaluate, and use digital information to make informed healthcare decisions. Inadequate DHL can also hinder effective physician–patient communication. The Smart Tablet Education for Healthy Living (STEHL) intervention leverages digital technology to enhance health literacy and promote healthy behaviors among older adults. This study aims to evaluate the impact of the STEHL intervention through qualitative insights from focus groups conducted with participants.

**Research Design and Methods:**

Three focus groups (n = 13) were conducted by trained facilitators in quiet rooms at three retirement homes in Illinois. Using a semi-structured interview approach, the focus groups explored participants’ experiences, perceptions, and behavioral changes resulting from the intervention. Responses from each question were imported into MAXQDA Analytics Pro 2024.

**Results:**

Thematic analysis revealed 12 themes and positive outcomes including increased confidence in using digital tools for health-related tasks, improved understanding of health information, and reported positive changes in lifestyle habits. Participants highlighted the intervention’s user-friendly design and personalized content as significant facilitators, while challenges such as initial apprehension toward technology were also noted.

**Discussion:**

These findings underscore the potential of the STEHL intervention to address health literacy gaps and support older adults in achieving better health outcomes. Future research should explore scalability and long-term effects of digital health interventions like STEHL in diverse populations.

